# Schizophrenic Consciousness in the Light of the Phenomenological Epoché: A Foundational Map for Psychiatry

**DOI:** 10.3390/brainsci16050498

**Published:** 2026-05-01

**Authors:** Giovanni Stanghellini

**Affiliations:** 1 Department of Health Sciences, University of Florence, 50139 Florence, Italy; giostan@libero.it; Tel.: +39-347-379-0707; 2 Centro de Estudios de Fenomenologia y Psiquiatrías, Diego Portales University, Santiago 8370068, Chile

**Keywords:** clinical phenomenology, epoché, hyperreflexivity, schizophrenic consciousness, unworlding, vital drive, neuropsychiatry

## Abstract

**Highlights:**

**What are the main findings?**

- This review illustrates that schizophrenic symptoms are not discrete, unrelated deficits, but rather an integrated whole, emerging from a fundamental reorganization of consciousness.
- It establishes a conceptual kinship between the methodological suspension of the natural attitude in philosophy and the passive, involuntary collapse of reality experienced in schizophrenia.
- It provides a Comparative Matrix as the necessary bridge between anomalies of consciousness and the concrete symptoms of contemporary psychiatric classification by aligning the various modes of the epoché/reduction in consciousness with standard DSM-5 symptoms.
- Specific clinical symptoms are mapped to phenomenological reductions: eidetic reduction manifests as delusional perception (loss of common-sense typifications), while transcendental reduction mirrors the hyperreflexivity of the “internal machinery” of the mind.
- Negative symptoms, such as flat affect and avolition, are redefined not as simple functional deficits, but as a collapse of the “vital drive” and the pragmatic “readiness-to-hand” of the world.

**What are the implications of the main findings?**

- Schizophrenic symptoms are reframed as an immanent possibility of the human condition rather than a mere alienation from reality, providing a shared ontological ground between patient and clinician.
- The paper advocates for a hermeneutically grounded mental state examination, transforming clinical diagnosis from objective labeling into an empathetic act of shared human understanding.
- One might think that a phenomenological study on the *epoché* is of little interest to neuroscience; however, a precise description of the “what” and “how” of experience is the essential foundation for investigating the “why” of its biological correlates. By shifting focus from “thin” surface symptoms to *core phenomena*, phenomenology provides a structurally accurate “map” that prevents from grouping clinically and biologically distinct conditions under the same behavioral labels. This review suggests that skipping this descriptive stage leads to faulty diagnostic and causal models, as nosography and neurobiology cannot successfully explain what it has not first accurately defined through a “thick” analysis of the structure of consciousness.
- Ultimately, identifying these core modifications offers a necessary interdisciplinary alignment between clinical phenomenology, neuroscientific research, psychiatric classifications and therapeutic practice.

**Abstract:**

This review explores the hypothesis that schizophrenic symptoms may be understood not as isolated deficits, but as interconnected manifestations of a structural reorganization of consciousness. The premises of this work are grounded in a comparative matrix that suggests an underlying “consanguinity” between the philosopher’s voluntary epoché—the suspension of the natural attitude performed to study the inner workings of consciousness—and the involuntary “unworlding” passively experienced in schizophrenia. By exploring this shared ontological ground, the text suggests how specific phenomenological shifts, such as the collapse of the “vital drive,” may manifest as clinical markers; this process may eventually lead to an involuntary “transcendental reduction” where the mind’s internal machinery becomes an object of forced awareness. Building on these premises, the review tentatively outlines several key achievements. It addresses the substrate-subjectivity gap by linking biological sensory-binding failures to the onset of involuntary hyper-reflexivity. Regarding structural loss and gain of function, it suggests that the psychotic transition involves a simultaneous erosion of common-sense coherence and an intensified receptivity to unfiltered perceptual fragments, which may trigger a search for metaphysical meanings. In terms of a therapeutic synthesis, it proposes exploring the conversion of “artless decentering” into a manageable, strategic distance through mindfulness and person-centered position-taking. Finally, it discusses a potential nosographic evolution, advocating for future diagnostic classifications that prioritize the experiencing self and qualitative insights to support a more translational and empathetic approach to psychiatry.

## 1. Introduction: The Phenomenological Method as a Bridge to Schizophrenic Experiences

This article suggests that the boundaries between philosophical reflection and pathological experience may be blurred. A compelling example is found in the autobiographical accounts of Elyn Saks, a philosopher living with schizophrenia, who describes the fragmentation of their reality in terms that closely mirror formal phenomenological analysis: “Philosophy and psychosis have more in common than many people (philosophers especially) might care to admit. The similarity is not what you might think—that philosophy and psychosis don’t have rules, and you’re tossed around the universe willy-nilly. On the contrary, each is governed by very strict rules. The trick is to discover what those rules are” [1].

This personal testimony provides the necessary context for the following discussion: the intersection between the phenomenological method and psychopathological experience reveals a profound analogy between the programmatic suspension of naïve belief in the existence of the world and in the significance of worldly entities performed by phenomenologists, on one side, and the involuntary lived experiences of severe derealization and depersonalization in persons with schizophrenia, on the other. The present analysis proposes that the “strict rules” philosopher Elyn Saks writes about may find a theoretical parallel in the phenomenological concepts of *epoché* and *reduction*. Literally, epoché means cessation (from Ancient Greek “to hold back,” “to pause”) and reduction means leading-back (from Latin “re-ducere”). These terms have multiple methodological meanings within the sole framework of Husserl’s phenomenology and even more meanings in the subsequent phenomenological tradition [2,3]. Yet, in phenomenological philosophy they principally mean a programmatic bracketing of the “natural attitude” that governs our everyday “commonsense” existence (epoché), leading to (reduction) the systematic disclosure of the mind-world inseparable correlation, that is, to the awareness that the apprehension of worldly entities is inextricably related to subjectivity—and more exactly to embodied subjectivity and intersubjectivity. In both the philosophical exercise and the schizophrenic condition, the familiarity and “taken-for-grantedness” of the world are obscured, leading to a state where strange experiences may occur, the embodied self loses its grip on the world, and consciousness may become aware of its implicit workings.

In this paper, I argue that the rigorous study of the phenomenological method of the epoché developed by philosophers and the careful unfolding of the world disclosed by it—a world reduced to a correlate of subjectivity—provides a crucial framework for clinicians to better describe, recognize, and understand the complex subjective experiences of persons living with schizophrenia. And yet, a substantial difference exists between the two: while the phenomenologist enters this state deliberately to achieve an epistemological vantage point, the person with schizophrenia involuntarily inhabits this new way of being in the world. While both the philosopher and the patient experience a suspension of common-sense experience, they are separated by the presence or absence of agentic insight. The philosopher performs a controlled reduction, tethered by a metacognitive anchor; the patient, however, is thrust into a fractured hermeneutic field where the shared meanings of the world dissolve, leaving them without the reflective tools necessary to integrate the experience.

Despite these significant differences, when viewed through the lens of the *epoché*, the experience of persons with schizophrenia may be understood, not only as a deficit or an abnormality or merely as a disorder of consciousness, but as an inversion of the traditional order of existence with its own intrinsic logic, following the philosophical maxim *primum philosophari*, *deinde vivere* (philosophizing first, living second). In persons with schizophrenia, ontological and epistemological concerns may take precedence over the mundane factuality of this [4]. Persons with schizophrenia are not satisfied with what appears in immediate experience and have a particular propensity for metaphysical ruminations and, at times, philosophical speculations [5,6,7]. By prioritizing the “philosophical”—the search for the hidden essence of reality and for the secret workings of the mind—over the “living”—the pragmatic engagement with everyday life—persons with schizophrenia may become primarily concerned with the fate of humanity or the discovery of a grander reality. For these persons, the living world of appearances is treated as a secondary concern, or even as an illusion to be transcended in favor of a deeper, revelatory truth [8].

To provide a clear and consistent path through this complex intersection of disciplines, this work is organized as follows. First, it explores the historical and autobiographical context of schizophrenic experience, using the insights of philosophers with lived experience to frame our inquiry. Second, it proposes a theoretical alignment between the structures of the phenomenological *epoché* and the structural modifications of psychotic consciousness. Third, these concepts are crystallized into a ‘Comparative Matrix’ that maps specific DSM-5 symptoms to their phenomenological underpinnings. Finally, this analysis concludes by examining the translational, nosographical, functional-structural and therapeutic implications of this map, discussing how these epistemological boundaries can inform contemporary neuroscience and reshape current psychopathological horizons.

### 1.1. Preliminary Remarks: On Clinical Method, the Dual Reality of Schizophrenia and the Dialectical Balance Between Generalization/Individual Variations

Before delving into the analogies between the epoché described by philosophers and the experience of people with schizophrenia, four preliminary points must be made.

First, in these preliminary remarks on clinical method, I argue that while a study of the *epoché* may appear to be a purely philosophical exercise, it in fact establishes the necessary ground for a more rigorous science. It links directly to contemporary neuropsychiatric debates regarding phenotype discovery and the validity of diagnostic constructs. To address the ‘dual reality’ of schizophrenia—where universal structural changes meet highly individual variations—we must move beyond a reliance on ‘thin’ surface symptoms. This review aims to propose that schizophrenic symptoms are not a collection of isolated deficits, but interconnected epiphenomena of a profound, underlying alteration of consciousness. By identifying the *core phenomena*, phenomenology provides a structurally accurate map that prevents the faulty diagnostic and causal modeling sometimes found in nosographic and neurobiological research. Far from being a descriptive luxury, capturing these deep structural modifications is a scientific necessity; it provides the essential framework for a truly explanatory, interdisciplinary psychopathology that respects both the general essence of the disorder and the unique lived reality of the individual.

Second, this paper develops a use of the phenomenological epoché that is distinct from the Jaspersian tradition [9] with which psychiatrists are in general more familiar. Jaspers utilizes the method of the epoché primarily to ensure descriptive fidelity. In that psychopathological framework, the epoché is treated as a tool for epistemic neutrality: it is only by suspending theoretical doctrines and pre-knowledge that the clinician can attempt to faithfully reproduce the patient’s experience in a detailed and holistic manner [10]. In this way, the Jaspersian approach anchors the clinician in the patient’s subjective narration to grasp “the psychopathological phenomena themselves” as they emerge in the clinical encounter. My development of the epoché in this paper, however, shifts the focus from this descriptive ideal toward a method that does not merely clear the path for the clinical encounter, but rather seeks to uncover the constitutive structures of consciousness that underlie the experience of people with schizophrenia.

Third, it is useful to clarify from the outset that the epoché experienced by the person with schizophrenia involves only a partial loss of their existential grip on the shared world. Despite the epoché, the individual continues to inhabit the common-sense world we all share while simultaneously dwelling in a “reduced” one. Without this co-presence of worlds there would be no ontological common ground, making the therapeutic encounter and the possibility of care impossible. This mode of existing astride two distinct realities—the common-sense world and the world reduced by the epoché—is known as the phenomenon of “double bookkeeping” [11,12,13]. Introduced by Eugen Bleuler [14] to describe a “double reality,” this term captures a fundamental and paradoxical feature of the schizophrenic condition: the fact that a psychotic reality can exist parallel to a shared reality, even when these realities seem mutually exclusive.

Fourth, in attempting to uncover the constitutive structures underlying schizophrenic experience, a potential risk is the over-universalization of experiences that are deeply heterogeneous. It is important to acknowledge that growing cross-cultural and empirical evidence—spanning neuroscience, psychology, and philosophy—suggests that structures of consciousness and selfhood may not be uniform across all individuals or cultures. The brain and the “pre-reflective” structures of experience are “ecocentric”, that is literally “wired” by cultural environments and shaped by the social niche a person occupies [15]. Consequently, any generalization in psychopathology must be viewed through a *dialectic balance*: while this paper identifies structural similarities common to the schizophrenic condition, it does not suggest a monolithic experience that supersedes the patient’s unique individuality. My objective is to draw attention to shared structural themes without disregarding the profound individual and cultural variations that define each person’s lived reality.

In the following paragraphs, I will describe the main types of epoché, with particular focus on those I term “cognitive” and “vital”.

### 1.2. The Cognitive Epoché: Bracketing “Straightforward Cognition”

In our daily lives, we operate within a “natural attitude.” We assume the world exists exactly as we see it and interact with things automatically. Phenomenology begins with a radical “unplugging” from this habit, a process called the epoché, or a methodical disengagement from the world of straightforward cognition [16]. This involves suspending belief in something, or at least not operating with certain a priori beliefs [2]. By hitting the “pause” button on our relationship with the world, we perform a reduction—a leading back of all data to the subjectivity that constitutes them. This produces two main outcomes:

#### 1.2.1. From Particulars to Essences

Eidetic Reduction: The eidetic reduction is a shift from the ‘fact’ to the ‘format.’ While we usually see a specific object—like a chair—this reduction leads us to the universal *eidos* or ‘essence’ (chair-hood) that allows us to recognize any chair at all. In everyday life, our ‘natural attitude’ automatically filters the world into these familiar categories. The eidetic reduction suspends this filter, revealing the hidden ‘blueprint’ of consciousness that usually stays in the background. It involves transitioning from an empirical discipline of facts to an “eidetic science” of universal invariants.

#### 1.2.2. From Objects to the Act of Perceiving

Transcendental Reduction: The transcendental reduction is a shift from the ‘what’ to the ‘how.’ Normally, we are absorbed in the *what*, i.e., the objects we perceive. This reduction involves a sort of ‘I-splitting’ [17], where one part of us steps back to watch *how* our own mind ‘sees’ and ‘makes sense’ of the world. Instead of looking at the exterior scene, we look at the interior process. In schizophrenia, this becomes an involuntary ‘hyperreflexivity,’ where the patient can no longer simply live in the world, but becomes a trapped spectator of their own mental machinery.

Ultimately, the epoché is not merely the “neutrality” demanded by positivism. It is a disciplined mindfulness toward the problem of knowledge, necessary to ground our “natural belief” in the world’s existence. By exhibiting the evidence behind this belief, the epoché fulfills the philosophical ideal of self-responsibility [18].

### 1.3. The Vital Epoché: Bracketing the “Drive to Live”

While the first approach pauses our thoughts about the world, a more radical version—proposed by Max Scheler—seeks to bracket the basic vital drive (*Lebensdrang*) [19,20,21]. In our everyday natural attitude, we do not just “think” the world is real; we “crave” its reality through a striving directedness [22]. We are constantly seeking confirmation that the world will continue to be real in the next moment. If we suspend this instinctive interest in reality-verification, the world undergoes a haunting transformation:

#### 1.3.1. The Loss of the Vital Grip: From a Vibrant World to a Ghostly Realm

The “vital drive” acts as the invisible energetic tether that imbues the world with a sense of “thickness” and reality. In the natural attitude, we do not merely perceive objects as cold data; we feel their “pulse” through an embodied resonance. However, when this vital drive is switched off, our pre-reflective “grip” on worldly things fades, and the “reality element” of experience is canceled. The result is what Max Scheler describes as a “perfectly adynamic world” [19]—a state where objects lose their affective power and their ability to “move” us. Without the vital tension that links the subject to the object, the environment transforms into a ghostly realm of disembodied and meaningless representations [23]. This atmospheric shift is captured masterfully in the metaphysical paintings of Giorgio de Chirico; his works depict a world where figures appear as shadows or mannequins because of a “halt of the vital rhythm of the universe” [24]. This illustrates that our sense of “belonging” to reality is not a conclusion reached through cognitive judgment or logic, but is rooted in a primal, drive-based, and embodied connection to the world. In the schizophrenic experience, this “unplugging” of the life-force leads to deep derealization, where the world remains visible but feels ontologically “dead” or artificial.

#### 1.3.2. The Loss of Readiness-to-Hand: From Practical Flow to Alien Objects

To inhabit a world humanly is to encounter it through “concern” and practical engagement, a concept Martin Heidegger defines through the structure of the “world-hood” of the world [25]. In our daily lives, we do not perceive the tools we use as detached, “standing-there” objects (an *objectum*); instead, we encounter them as “equipment.” For instance, when we are absorbed in writing, the pen or the keyboard effectively disappears into the flow of our thoughts; we do not “see” the ink or the plastic keys as separate entities, but rather as transparent extensions of our own intentions. This is the state of being ready-to-hand.

However, when the involuntary epoché “unplugs” the patient from this practical immersion, the “handles” of the world—the intuitive meanings that allow us to navigate life without hesitation—suddenly fall away. When things are no longer “ready-to-hand,” they become “present-at-hand”: isolated, stark, and strange. A chair is no longer a familiar invitation to rest or a silent partner to the body; stripped of its functional essence, it is reduced to a purposeless geometric solid, a terrifyingly alien configuration of wood and fabric. For the schizophrenic patient, the world ceases to be a coherent “home” of useful tools and becomes a warehouse of disconnected “things” that must be painstakingly inspected because their obvious purpose has evaporated.

By combining these two brackets, phenomenology shows that our “reality” is built on both intellectual structures and biological drives. When one of them or both are paused, the network of practical and cognitive references that makes our world feel “home-like” vanishes, leaving us to examine how the sense of reality is built in the first place (for a synthesis of the various kinds of epoché see Table 1).

## 2. Schizophrenic Phenomena as Consequences of Involuntary Epoché

Phenomenological philosophers do not have the explicit intention of describing the experiences of persons with schizophrenia. And yet, their writings—dating back to the early twentieth century—have contributed enormously to the progress of knowledge within the field of psychopathology [26]. This contribution is particularly valuable in the context of studies on schizophrenia [27,28,29,30].

For persons with schizophrenia, the epoché is not a philosophical choice but an existential fate—an involuntary entry into a reduced world [31]. Several aspects of the schizophrenic condition reflect the epoché’s described by philosophers. In the following analysis, we trace the ‘thin’ symptoms defined by contemporary clinical manuals [32] back to their ‘thick’ phenomenological foundations—*core phenomena*—demonstrating how these clinical markers emerge as necessary consequences of the various forms of involuntary epoché.

*“Perplexity/Delusional mood”*. Delusional mood is defined as an ominous feeling that the world has changed and that something momentous is about to happen. In the words of one patient: “The air feels heavy, expectant. The streetlamp looks alien, glowing with a terrifying, secret meaning I cannot yet grasp.” Merleau-Ponty describes the reduction as a state of “wonder” achieved by stepping back from the world to observe it as a mystery. By purposefully slackening the intentional threads that bind us to reality, the epoché makes the world appear strange and paradoxical. This detachment is what allows the phenomenologist to truly notice the world, watching meanings “fly up like sparks” ([23], p. XV) rather than taking them for granted. In persons with schizophrenia, this slackening of intentional threads is not a meditative act of wonder, but a catastrophic unraveling of the world. While the philosopher intentionally loosens these threads to gain clarity, the patient experiences an involuntary snapping of the cords that anchor the self to reality. This transforms Merleau-Ponty’s sparks from a fire into a terrifying, enigmatic light where the most mundane objects become “strange and paradoxical”. “Wonder” becomes the “prodromal perplexity” and the “delusional mood” of the patient [33]. In this state, the world loses its familiar texture.

*“Delusional perception”*: Delusional perception is a primary experience where a correctly perceived object is suddenly imbued with a private, absolute meaning. This state is voiced as follows: “I saw a simple red car. Suddenly, I knew it wasn’t just a vehicle; it was a divine signal that I must save the world from an approaching, invisible apocalypse.” It mirrors a pathological version of the eidetic reduction: while the philosopher intentionally suspends common-sense “typifications” to let things “speak for themselves,” the schizophrenic patient undergoes an involuntary rupture of this balance [34]. Stripped of the “natural attitude,” the patient is overwhelmed by aberrant salience [35]—an unmediated, alien reality where mundane objects lose their familiar context [29]. In this state, the sensory data may remain accurate, but the shared intentional meaning is replaced by an apophanous revelation of “hidden,” self-referential truths [36]. The weight of experience shifts from mundane factuality toward a preoccupation with ontological, eschatological, and epistemological concerns [4,8]. Everyday matters are often discarded as the person becomes absorbed in the overarching meaning of existence or the ultimate fate of humanity. This is frequently seen in epistemological delusions—the sense of having uncovered the hidden essence of reality, leading the individual to view others as being trapped in a state of ignorance, aware only of mere appearances [7]. The epoché is also mirrored in eschatological delusions, where the person experiences a sudden transition into a grander, novel state of reality in which every phenomenon is imbued with a new, revelatory significance. Such experiences are often coupled with charismatic delusions—for instance, the conviction that one must save a world on the brink of collapse.

*“Disorganized Thinking/Thought Monitoring”*: Disorganized thinking is a cognitive pattern where, seen from a third-person perspective, thoughts are fragmented, illogical, or incoherent. To move beyond the third-person observation of ‘disorganized thought,’ we must consider the patient’s own description of their internal mechanics. A patient says: “My thoughts are no longer mine. They move like heavy, rattling gears I can see and hear, stalling before they ever reach my lips.” This can in part be understood as a consequence of a cognitive shift reminiscent of the transcendental reduction. Rather than being absorbed in the world, the person undergoes a conversion from the exterior to the interior, making the acts of consciousness themselves the objects of reflection. This is obviously a disconcerting experience that may trouble logical train of thought and disrupt effective communication. What Husserl intended as an epistemological tool to bring “constitutive subjectivity” into view becomes, in schizophrenia, a radical and involuntary experience of hyper-reflexivity [5,37,38]—a state of exaggerated self-consciousness where the “background” of experience-normally lived through automatically becomes an object of focal awareness. Mental processes that should be “invisible” become solid, strange, and “thing-like.” The self divides into a detached spectator and an observed object, leading to a loss of spontaneity and a “ghostly” sense of reality.

*“Depersonalization/Derealization”*: These are defined as dissociative phenomena characterized by a subjective feeling of detachment from oneself and the world. One of the unifying themes of schizophrenic existence—mirroring the Schelerian epochè—is a profound disembodiment, a structural collapse of the “lived body” as the primary vehicle for inhabiting the world [39]. This has several clinical implications. One of them, is a form of depersonalization—the patient feels like a “disembodied spirit” contemplating its own existence from a third-person perspective or a “view from nowhere”. Another is a characteristic form of derealization: the patient is transported into a world where objects and people appear unreal, indistinguishable from images or “cardboard cutouts,” and space itself resembles a theatrical set [40]. The patient loses vital contact with reality; everything appears in an enigmatic light, and it seems impossible to gain an effective ‘grip’ on the world. The cancelation of practical meanings, the divorce from things and their ready-to-hand meanings, is described in these passages from the *Autobiography of a Schizophrenic Girl*: “When, for example, I looked at a chair or a jug, I thought not of their use or function—a jug not as something to hold water and milk, a chair not as something to sit in—but as having lost their names, their functions and meanings [41]. I attempted to escape their hold by calling out their names. I said, “chair, jug, table, it is a chair”. But the word echoed hollowly, deprived of all meaning; it had left the object, was divorced from it, so much that on one hand it was a living, mocking thing, on the other, a name, robbed of sense, an envelope emptied of content” [41] (p. 56).

*“Social/Occupational Impairment”*: Social dysfunction is universally considered a basic diagnostic criterion of schizophrenia. “The subjective correlate of this diagnostic criterion is illustrated by the following self-report: “I am watching them laugh, but I only see teeth and hear noise. I must manually calculate their next move.” According to phenomenology, social functioning is based on intersubjectivity—the ability to understand other persons, to grasp the meaningfulness of their actions and expressions [42]. Intersubjectivity is first and foremost intercorporeality or inter-bodily resonance/attunement—a process of embodied engagement and coordinated interaction between one’s and others’ bodies that provides the basis for the intuitive understanding of the other person’s feelings and intentions [43,44]. The Schelerian epoché and the crisis of embodiment lead to a crisis of intercorporeal attunement [45]. When this resonance between bodies fails, the social world turns into a “cool, incomprehensible game” governed by abstract algorithms rather than emotional flow [42] which, in current diagnostic manuals, are named asociality/flat affect.

*“Cognitive errors” in the formation of delusions*: Handbooks define delusions as fixed false beliefs—a failure of logic or “top-down” data processing. However, a phenomenological approach suggests that delusions are actually secondary attempts to make sense of a primary existential collapse. Our sense of “reality” depends on the vital drive: we feel the world is real because it offers resistance to our actions. In schizophrenia, this drive is “switched off” (an involuntary epoché), causing the world to lose its density and “grip.” In this “bodiless” state, language shatters. The symbolic “as-if” of metaphor, used to express quasi-ineffable sensations, evaporates. This existential shift is vividly captured in the words of one patient, who describes the world as follows: “The world has turned to paper and I can read it one page after the other.” “When I say my heart is ‘shattered,’ I can feel the sharp glass shards cutting my chest.” Language escapes its shared, lived context and becomes a set of “objects” rather than tools. Figures of speech lose their distance and “become flesh”—a “broken heart” is no longer a symbol of grief, but a literal, physical fracture [46,47]. Delusions are not a “wrong thought,” but a desperate effort to anchor a consciousness that has lost its vital connection to a resistant, shared world [48] (for a comparative matrix bridging phenomenological epoché/reductions and DMS clinical symptoms see Table 2).

## 3. Identifying Phenomenological Reductions in the Mental State Examination: A Guide for Beginners

The analogy between the phenomenological epoché and schizophrenic consciousness suggests that the “reduced world” is a shared territory. Whether reached through Husserl’s cognitive bracketing and the loss of commonsense meanings or Scheler’s suspension of vital drives and the halting of our instinctive interest in proving the world is real—the result is a world where the “natural attitude” has collapsed. In this space, the clinician finds a unique vantage point: by understanding the epoché as a philosophical exercise, one can begin to intuit the “adynamic” and “ghostly” world inhabited by the patient, moving from a mere classification of symptoms to a poetics of recognition.

It is essential to recognize that the epoché performed by phenomenologists is a possibility immanent to human existence—not a form of psychopathological alienation devoid of any relationship to the humana condicio. Every human being, not only those with schizophrenia, can access experiences such as the splitting of the “I” into an observing subject and an observed object, or the profound wonder in the face of an enigmatic reality. This “consanguinity” between the methodological practices of phenomenologists and the lived experiences endured—and at times, at least in part, actively sought [39]—by people with schizophrenia serves as clear evidence that schizophrenia is not an external “brokenness,” but a variant of the human condition itself.

The phenomenological epoché serves as a diagnostic “lens” that allows the clinician to move beneath the surface of symptomatic reports. Ultimately, the clinical utility of this approach lies in the shift from a detached, third-person diagnosis to a shared, first-person understanding. If the clinician is willing to perform the epoché themselves, they can access experiences analogous to those lived by the patients, transforming a cold clinical encounter into an act of profound intercorporeal resonance. By recognizing the intrinsic logic within the “catastrophic unraveling” of the world, the clinician no longer sees a mere deficit or abnormality, but a human consciousness struggling to find meaning within the hidden folds of existence.

By understanding the diverse modes of the epoché the clinician can identify the specific structural displacement in the patient’s consciousness. This knowledge is useful because it provides a differential typology of experience: it helps the clinician distinguish between a patient who is merely “withdrawn” (a behavioral observation) and one who is inhabiting a Schelerian adynamic world, where the very “urgency of life” that anchors us to reality has been extinguished.

Even if the structural transformations of consciousness described here do not represent the totality of schizophrenic experiences, they can nonetheless provide a map and points of reference for the clinical interview. Integrating these pluralities into the Mental State Examination (MSE) transforms the assessment from a passive “accounting” of deficits into a sophisticated “mapping” of the patient’s existential architecture. Rather than recording “flat affect” as a static trait, the clinician recognizes a loss of readiness-to-hand, where the world has been reduced to a meaningless, geometric “sum of realia.” This awareness provides a robust epistemic foundation for the MSE, ensuring that the “disembodied world” of the schizophrenic experience is not merely categorized as “disorganized,” but understood as a profound, involuntary suspension of the natural attitude. Consequently, the phenomenological interview becomes an act of epistemic and hermeneutic justice [49,50,51]. By acknowledging that the patient’s “disembodied spirit” or “view from nowhere” is a radicalized version of a shared human potential, the clinician moves beyond mere diagnosis toward an ethical recognition of the patient’s world. Integrating the epoché into the MSE allows us to validate the patient’s ghostly, adynamic reality as a structural possibility of being, thereby transforming the clinical encounter into a poetics of recognition. This guide is designed to help clinicians (including trainees or “beginners”) translate the complex theoretical concepts of the Husserlian and Schelerian epoché’s into practical observations during the MSE. By identifying these “philosophical markers,” the clinician can move from a third-person observation of symptoms to a second-person understanding of the patient’s lived world.

### 3.1. Identifying the Eidetic Reduction (The Shift to Hidden Meanings)

In the context of MSE, the principal character of the eidetic reduction is the crisis of the “quality of familiarity” [52]—the “commonsense” typifications of the world are suspended, the patient is left in a state of exalted receptiveness, where the taken-for-granted meanings that usually “cloak” objects are stripped away. The patient feels like a passive receptor of a “hidden” or “essential” meaning that seems to emanate directly from the things themselves, unmediated by social or cultural context.

*Nosological Context*: typical in early-stage schizophrenia or acute psychotic crises.

*Clinical Marker*: Delusional Perception. A normal perception is suddenly imbued with a self-evident, urgent, and private significance that reveals the “true essence” of the situation.

*What to Listen For*: Objects or events appear to the patient “unfamiliar”, “different”, or “intense” or “salient”. This is followed by an undeniable epiphany. For example: “The way the three pens were laid out on the desk wasn’t just a coincidence; it was a sign specifically for me that the trinity is watching.”

*The Patient’s Experience*: The world “speaks” directly to them. Objects lose their mundane function and reveal a “hidden character” or an absolute truth that was previously concealed by the “prejudices” of everyday life. The veil of Maya has been torn. Example: “The streetlights didn’t just turn on; their sequence was a coded script, revealing the absolute geometry of my fate.”

*Inquiry Strategy*: Focus on the quality of the perception. Ask: “Did the object itself seem to change its meaning for you? Did it feel like the object was ‘disclosing’ a secret or a message that was already there, waiting to be seen?”, “What did you learn from this?”.

*Differential phenomenology*: This must be distinguished from other types of delusions like holothymic delusions in manic-depressive psychoses [53] which are not characterized by revelation (the sudden manifestation of a meaningfulness that is normally hidden) [29], but rather structured as the validation of a belief that the person already has about themselves that is already implicit in their value system [54].

### 3.2. Identifying the Transcendental Reduction (The Shift to Noetic Processes)

In the transcendental reduction mode, the patients are hyper-aware of the process of experiencing external (i.e., perceptions) and internal (i.e., thoughts and emotions) phenomena. The mind mirrors its own mechanics.

*Nosological Context*: this is often seen during the whole course of schizophrenia or high-functioning schizotypy.

*Clinical Marker*: Hyper-reflexivity. The patient describes the “mechanics” of their thoughts rather than the content.

*What to Listen For*: “I can feel the words being formed in my mind before I say them,” or “I am watching myself watch the television.”

*The patient’s experience*: The patient has “unplugged” from the natural flow of life to observe their acts of thinking or perceiving. Example: “I am not thinking my thoughts; I am standing behind them, watching the gears of my mind slowly turn and click.”

*Inquiry Strategy*: Ask: “Do you feel like you are a participant in your thoughts, or a detached observer of how they are made?”

*Differential phenomenology*: To be distinguished from obsessive thinking—recurrent, intrusive, and unwanted “contents” like thoughts, urges, or images—not the monitoring of noetic processes [55].

### 3.3. Identifying the Loss of Vital Grip with Reality (The Shift to a Ghostly World)

When the *Lebensdrang* (urgency of life) is “switched off,” the world becomes a “ghostly” or “metaphysical” place. The patient loses the vital drives that glue the self to the world—a sense of being laid bare that might be termed a mute apocalypse.

*Nosological Context*: common in so-called “negative” or “pauci-symptomatic” forms of schizophrenia.

*Clinical Marker*: Adynamic Worldview. The patient describes a world that feels “flat,” “cinematic,” or “unreal.” Things look like props in a play. To be distinguished from depressive apathy.

*What to Listen For*: “The world looks like a painting,” or “Everything is made of cardboard; it has no weight or effect.”

*The Patient’s Experience*: The patient has lost the sense of reality. No action is meaningful or effective anymore. People seem to behave like in the movie “The Truman Show”. Example: “I am walking through a movie set. The buildings are just cardboard facades, and the people are actors following a script that has nothing to do with me.”

*Inquiry Strategy*: Ask: “Does the world around you feel like a solid place where you can act, or does it feel like a series of disembodied images or shadows?”

*Differential phenomenology*: To be distinguished from depressive avolition-apathy (typically accompanied by intense sorrow/guilt feelings) or neurological adynamia (inability to start/finishing tasks, reduced spontaneous actions) [56].

### 3.4. Identifying the Breakdown of Readiness-to-Hand (The Shift to a Meaningless World)

This is the most “instrumental” marker. It occurs when the “readiness-to-hand” of the world collapses into a purely theoretical or geometric presence.

*Nosological Context*: typical in pre-schizophrenic conditions characterized by the pre-delusional atmosphere (Wahnstimmung) but also in so called “pauci-symptomatic” or so-called “negative” forms of schizophrenia (where no “positive” symptoms are in the foreground).

*Clinical Marker*: De-pragmatization of Objects. The patient treats familiar objects as strange, geometric entities rather than useful tools.

*What to Listen For*: A patient might stare at a fork and describe it as “four silver tines reflecting light in a parallel extension” rather than “something to eat with.”

*The Patient’s experience*: The patient sees the world as a “sum of realia” in a geometric void. The “in-order-to” quality of life has vanished. Example: “I stared at the spoon for an hour. It was just a curved, metallic surface reflecting a distorted version of the room.”

*Inquiry Strategy*: Notice if the patient handles objects with hesitation. Ask: “When you look at a common object, like a chair, do you see its use, or do you see a strange shape that just happens to be there?”

*Differential phenomenology*: To be distinguished from depersonalization/derealization disorder where the patients says “It is as if” or “I feel like” [55,56].

### 3.5. Identifying Radical Disembodiment (The Shift to Full-Blown Delusional World)

Disembodiment is a hallmark experience in schizophrenia [39]. At its most basic level, it consists of the feeling that one’s self—including one’s mental functions—is no longer rooted in the body. This experience of being a “disembodied self” profoundly alters how a person encounters the world. The ultimate result of this process is perfectly captured by a patient’s words: “I can’t distinguish between thought, imagination, and reality.” For a disembodied spirit, the usual boundaries of reality begin to blur. Thoughts become things: A person’s thoughts can take on the density and material “solidity” of a physical object in the room. Things become images: Conversely, a physical object may lose its depth and tridimensionality, appearing as a mere immaterial representation, a flat painting, or a cardboard cutout. Words become flesh: Even language can “take body” and be experienced as a concrete thing. This collapse of boundaries is epitomized by the Surrealist painting of René Magritte. A striking example is his work Le Coup au Cœur (The Blow to the Heart), which depicts a rose with a stem transformed into an enormous, dagger-shaped thorn. In this world, the boundary between the “idea” of a thorn and the “reality” of a weapon that wounds the heart has dissolved. Ultimately, in the schizophrenic experience, the distinctions between thoughts, words, and concrete things fade away into a single, flattened plane of existence.

*Nosological Context*: Disembodiment can be the “gate” through which people with schizophrenia enter to full-blown delusional world.

*Clinical Marker*: Semantic Collapse and Literalism. The images used to signify a feeling or sensation are no longer metaphorical descriptions; they are lived as a literal, tangible fact. Because the vital connection to the world is lost, the “as if” evaporates: the figurative becomes literal.

*What to Listen For*: The patient says “My heart is a stone,” and genuinely worries about the mineral weight in their chest, they have lost the symbolic distance that embodiment provides.

*The Patient’s Experience*: To distinguish between things, words and images may become extremely difficult. Words (metaphors) may become literalized, or worldly things may be experienced as mere representations or “cardboards”. A shift to pure logic (morbid rationalism) rather than pragmatic concerns may happen. Example: “My thoughts are as solid as bricks, and my heart is a literal stone, heavy and cold, sinking into my stomach.”

*Inquiry Strategy*: Notice if metaphors are no longer a symbol of a feeling, but a concrete physical reality. Listen for “disembodied values”—ethics or morals that are perfectly logical but devoid of human warmth or social situatedness.

*Differential phenomenology*: This is to be distinguished from anomalous bodily phenomena in persons with major depression who experience their body as a dead “thing” lacking vitality; their body becomes a burden, an obstacle that hinders natural engagement in the lived environment [57].

## 4. Toward a Phenomenologically Informed Clinical Practice

Comparing the schizophrenic experience to the phenomenological practice of epoché explicates that the schizophrenic form of consciousness is not an alien state, but rather a variant of the human condition. The primary task of psychopathological knowledge is to recognize the “human” within the pathology. It is therefore essential to understand the root of schizophrenic symptoms as involuntary modifications of consciousness—states that mirror the very same voluntary shifts in awareness performed by philosophers to uncover the implicit structures of the human mind.

Phenomenology’s ability to grasp and help us comprehend the lived experience of people with schizophrenia stems from its capacity to uncover the very structure of human existence. The “I-splitting” is a fundamental phenomenon in human consciousness [17] and in schizophrenic consciousness [14]. The splitting of the Self into an observing part and an observed part—epitomized in the schizophrenic condition—is an intrinsic possibility of human consciousness. Although more attenuated than in schizophrenia, this splitting is realized in human existence in the phenomenon of self-reflection, during which one part of the Self turns toward itself to make its own thought processes and beliefs explicit, allowing for critical examination. Schizophrenic consciousness pushes this process to its extreme and to a point of pathological torsion; it does not implement a thought process that is entirely alien to the human condition.

This comparative analysis seeks to show that the schizophrenic experience is not merely a collection of isolated deficits, but a coherent—albeit devastating—structural transformation of human consciousness. The transition from a healthy “natural attitude” to a state of radical cancelation of commonsense cognition, switching off of the vital drive and finally disembodiment represents a catastrophic shift. Where phenomenology sought the eidetic and transcendental reductions to reach the “things themselves” and the machinery of thought, schizophrenic patients may suffer these reductions as an involuntary fate. The world is no longer a shared “workshop” of shared meanings. In this reduced state, the patient inhabits a reality where hidden and revelatory meanings “pop up” from the environment, and consciousness—stripped of its outward engagement—is forced to focus on itself rather than on the world.

Through the lens of Scheler, we see that the epoché is far more than a cognitive recalibration or a suspension of belief; it is a vital amputation. It does not merely strip away our commonsense forms of typifications and intellectual prejudices but cancels the very *Lebensdrang* that allows a thought to become an act. The loss of the vital drive severs the embodied foundation of cognition. When the “call to action” is extinguished in persons with schizophrenia, they lose their “grip” on the world, and language loses its symbolic distance, causing metaphors to “become flesh.” This catachresis is the final marker of a consciousness that has bracketed the world so thoroughly that it can no longer inhabit it.

By charting the patient’s experience against the pluralities of the phenomenological epoché, we move beyond a purely descriptive accounting of symptoms toward a “mapping” of existential architecture. Drawing upon the rich and detailed descriptions of the epoché’s provided by philosophers can help “give a voice” to patients who, overwhelmed by the psychotic experience, find themselves without the words to articulate it. This approach fosters a therapeutic dialogue grounded in the clinician’s own phenomenological expertise and potentially on their own phenomenological experience—an experience placed at the patient’s disposal to facilitate the vital work of self-recognition and meaning-making.

Ultimately, integrating the epoché into the MSE is an act of hermeneutic justice. It allows the clinician to recognize that the “view from nowhere” or the “disembodied spirit” is not a sign of disorganized thought, but a structural reality, a form of existence with its own meaning. By using the phenomenological periscope, the clinician transforms the clinical encounter into a poetics of recognition—validating the patient’s ghostly, adynamic world while refining the diagnostic precision necessary for modern practice.

## 5. Discussion and Implications: Bridging Phenomenology, Neuroscience, Nosography and Therapy

Having established the “Comparative Matrix” and mapped the structural parallels between the phenomenological *epoché* and schizophrenic experience, it is now possible to address the broader translational, nosographical, functional-structural and therapeutic implications of this foundational map.

One might think that a phenomenological study on the effects of the *epoché* would be of little interest to the field of neuroscience; yet, to properly explain a psychopathological phenomenon—including looking for its neurobiological correlates—researchers must first shift their focus from “surface” signs to a “deep” phenomenological description, as a precise understanding of the “what” and the “how” of experience is the only valid foundation for investigating the “why” of its causes. Phenomenology is not a substitute for neuroscience, but a constraining condition. This ensures that neurobiological models are targeting the structural essence of the disorder rather than just surface-level epiphenomena. In neuroscientific research, the prevailing reliance on surface symptoms (such as social withdrawal or flat affect) is often justified by their high inter-rater reliability; however, this consistency comes at the expense of validity, as these “thin” descriptions often group together biologically distinct conditions under a single behavioral label. For instance, the surface sign of “withdrawal” in depression typically stems from anhedonia and a lack of vital energy, whereas in schizophrenia it may emerge from an ipseity-disturbance or disorder of self-world attunement where reality itself feels “unreal.” By employing careful phenomenological analysis to identify the *core phenomena* or the “formal cause” of a condition, we provide neuroscience with a structurally accurate “map” of the pathology. This deeper analysis allows for the identification of both synchronic relationships (how different symptoms imply and necessitate one another) and diachronic processes (how core disturbances evolve over time into compensatory behaviors), moving beyond mere epiphenomena to target the actual structural essence of the disorder. Ultimately, as modern psychopathological discourse suggests, neurobiology cannot successfully explain what it has not first accurately described; therefore, the development of informative, “thick” symptoms through phenomenology is not a descriptive luxury, but a scientific necessity for a truly explanatory psychopathology.

Before attempting to explain *why* something happens (causal explanation), one must first thoroughly understand *what* is happening. This involves a neutral, detailed description of the experience or phenomenon itself. A proper, detailed description acts as an epistemologically necessary “constraining condition” on any subsequent explanation. An explanation that contradicts the observed phenomena is inherently flawed. This approach argues against jumping to premature conclusions or causal mechanisms before the phenomenon is fully understood or captured, a common pitfall in science.

In summary, I suggest with this paper that skipping the descriptive stage leads to faulty explanations and systems, emphasizing that understanding the structure of the phenomenon is fundamental to explaining its causes or biological correlates. The ‘heart’ of this analysis consists of attempting to richly describe the patients with schizophrenia’s lived experience in detail and tracing these experiences back to structural modifications of consciousness. In this way, it suggests a series of interdisciplinary tensions without, however, developing them extensively. Without assuming a seamless convergence, it shows a potential alignment between clinical phenomenology, psychopathological assessment/diagnosis, neuroscience and therapeutic practice:

### 5.1. The Substrate-Subjectivity Gap

This paper pursues two primary, harmonized objectives.

(1) Clinical/Holistic: the primary goal is to provide clinicians with a unified, holistic framework to navigate the “complexities” of schizophrenic symptoms. By using the “lens” of the epoché, we can move beyond a fragmented checklist of symptoms to a coherent understanding of the patient’s lived reality.
(2) Neuro-phenomenal: Complementary to the clinical aim, the paper seeks to provide neuroscientific researchers with a precisely defined “core disorder”. Following the neurophenomenological tradition, the “neuro-” prefix signifies a commitment to the idea that structural modifications of consciousness are the formal substrate for which neurobiology must seek a correlate. Without this descriptive “map,” biological research risks targeting surface epiphenomena rather than the core of the pathology. The term ‘neurophenomenal’ is employed here in the strict sense proposed by Varela [58], wherein first-person descriptions and neurobiological data serve as reciprocal constraints. This approach follows the enactive framework, e.g., [59,60,61,62,63], which argues that mental processes cannot be understood without a rigorous analysis of the structures of lived experience.

The epoché entails anomalies of consciousness. To bridge the gap between the neurobiological and the phenomenal (*Bewusstseinsstörungen* = disorders of consciousness), we can place Gerd Huber’s work as the foundational link. Huber’s “basic symptoms” [64] serve as the bridge between brain and experience where the “organic substrate” first begins to tremor, manifesting as the “substrate-close” subjective phenomena precursors to both the loss of grip and the onset of pathological hyper-reflexivity. The basic symptoms of schizophrenia are defined as subtle, subclinical, and subjectively experienced disturbances in mental processes—such as “thought interference,” sensory hypersensitivity, cenesthesias, or diminished “vital drive”—that are not yet overtly psychotic but represent the primary “substrate-close” epiphenomena of brain instability. These symptoms are viewed as the building blocks of the pathology. The integration of Huber’s findings with contemporary neuro-phenomenology suggests that the “flickering” of the brain substrate may be linked to an involuntary version of the crisis of vital drive and transcendental reduction. In a healthy state, the “natural attitude” is maintained by a robust vital drive—the Huber-defined energetic baseline that allows us to inhabit the world without effort. When this vital drive is reduced, connected to brain instability, the individual loses their “grip” on the familiar; the world is no longer an arena of invitations and actions, but a distant, flattened landscape of “reduced” phenomena. This loss of grip is a symptom of the failure of the automatic integration of sensory data. The “dys-integration” of the senses leads to three interconnected disruptions in how a person experiences reality: (a) loss of the basic, automatic feeling of being the center of one’s own experiences (the feeling of being traversed by uncoordinated flows of perceptions and bodily sensations), (b) loss of “grip” on the world (a) feeling that the external environment has lost its familiarity, stability, or meaning, (c) operative hyperreflexivity (a) state where things that should be automatic, like walking or thinking, suddenly become objects of intense, forced conscious awareness. When perceptual disintegration occurs, the person undergoes a forced transcendental reduction, where the “bracketed” world becomes an object of terrifying alien study rather than a lived reality.

An engaging hypothesis linking the phenomenal level to the pre-phenomenal (neurobiological) level is as follows: at the heart of this hypothesis is the idea that the “flickering” of the brain substrate is the brain’s failure to achieve “temporal binding”—the seamless blending of sight, sound, and internal proprioception into a unified whole [65,66,67]. Perceptual dys-integration triggers an involuntary shift in the very structure of human experience. In Huber’s model, the “organic substrate” is the biological foundation for the vital drive (*energetisches Potenzial*), the basic force that sustains our unthinking immersion in the world. When this substrate becomes unstable, manifesting as “basic symptoms” (varieties of perceptual dys-integration including, e.g., cenesthesias, thought interference, anomalies of perception, etc.), the natural attitude begins to collapse. This collapse corresponds to Scheler’s reduction in vital drive and is experienced as a loss of “grip”—the world loses its practical familiarity and its “affordances” for action. Consequently, the person undergoes an involuntary transcendental reduction: because the brain can no longer automatically “bind” sensory inputs, the person is forced into a state of operative hyper-reflexivity. This hyper-reflexivity is the mind’s desperate attempt to intellectually reconstruct a reality that is no longer being provided by the failing substrate. Thus, the hyper-reflexive gaze is a desperate, secondary attempt to re-establish a sense of self-presence in the wake of a crumbling vital foundation. In this state, the diminished self-presence (the loss of the “I-me-mine” feeling) forces the individual to observe their own internal processes—thoughts, movements, and sensations—as if they were external, alien objects. Thus, the “Substrate-Subjectivity Gap” is bridged by a tragic irony: the more the biological substrate fails to integrate the world, the more the person is forced into a hyper-conscious, “reduced” state of existence, forever watching the machinery of a self they no longer fully inhabit. To be sure, research bridging phenomenology and neuroscience still has a long way to go, but at the very least, mutual prejudices have faded, and the paths seem to be beginning to converge [68].

### 5.2. Structural Loss and Gain of Function

This review illustrates that the transition into psychotic consciousness is not merely a deficit, but a complex reorganization characterized by a simultaneous loss and gain of function. The anomalies of consciousness connected to the various forms of epoché involve the attenuation of “common-sense” world-, body-, and self-experience—the stable, subjective/intersubjective “priors” that constitute our habitual reality. Crucially, this process entails a simultaneous *loss* and *gain* of function. Loss of function: the erosion of global contextual coherence and the “natural evidence” of daily life. Gain of function: a state of hyper-reflexivity (as explained in the previous section) and an intensification of sensory data. The “gain” of the schizophrenic consciousness after the epoché consists of a heightened emotional sensitivity, an exceptional receptivity to “atmospheres,” an extraordinary perceptual acuity, especially for details, and a particular responsiveness to that which remains “invisible” to the normal consciousness—that is, to the enigmatic, the arcane, the “double,” and the mysterious. Our “normal” consciousness, instead of merely dismissing these divergent faculties of the schizophrenic consciousness, should learn to value their ability to bring to light the hidden folds of our habitual, familiar experience of reality. This phenomenological “bracketing” aligns significantly with modern models of aberrant salience [35] and predictive processing [69]. In these models, phenomenal instability leads to an over-reliance on raw sensory input at the expense of top-down contextual integration. From this perspective, the outcome of the schizophrenic *epoché* is not a vacuum of experience, but rather an agonizingly crowded field of unmediated perception corresponding to systemic neurophenomenal reconfiguration. Validating this hypothesis further will require rigorous empirical studies [70].

### 5.3. Therapeutic Synthesis

The core therapeutic challenge lies in converting the epoché—an involuntary decentering from one’s self—into a manageable distance from one’s own self. In this sense, persons with schizophrenia are, so to speak, “artlessly decentered.” In this context, the therapeutic objective is not necessarily a forced return to a lost natural “centric” attitude, but rather the cultivation of a strategic distance from anomalous self- and world-experiences. This alignment is central to mindfulness and to the person-centered dialectical model. The *epoché* in persons with schizophrenia, manifesting as an involuntary and distressing collapse of the natural attitude, bears a striking resemblance to the kind of voluntary *epoché* practiced in advanced meditative states. Neurophenomenological research [71] suggests that mindfulness training involves a form of *decentration*—a shift away from habitual self-contact toward an equanimous, unmediated perception of stimuli [72]. By adapting mindfulness, clinicians provide a framework for “decentering” from the self. This allows the patient to observe anomalous experiences not as absolute reality, but as mental events. Similarly, in the phenomenological person-centered dialectical model, “position-taking” [73] is a methodology focused on transforming the patient’s stance regarding their basic experiential anomalies. Position-taking is a dynamical construct; it entails the patient’s active “working-through” of anomalous experiences facilitated by a distancing stance. Both mindfulness and position-taking enhance a deliberate reflexivity toward basic anomalies of experience. This active distancing liberates the cognitive space necessary to seek meaning, cause, or reason—enabling patients to evaluate the significance of these experiences within their life trajectory and gain insight into their pathological or idiosyncratic phenomena. By framing the patient’s journey through the dual lenses of mindfulness-based decentering and the person-centered dialectical model, clinicians can help patients navigate their “bracketed” world with greater intentionality and less catastrophic disintegration [74].

### 5.4. Clinical Directions and Nosographic Evolution

In light of this paper, the emphasis on the “experiencing self” should be given the relevance it deserves, and future nosographic categorizations of schizophrenia must take this dimension into account—as it is the case with ICD-11 [75]. Furthermore, clinical and research protocols should prioritize “top-down” strategies that allow clinicians to grasp the profound importance of the pivotal role of insight and of the patient’s position-taking with respect to their basic disorders of experience. Rather than focusing solely on the “bottom-up” flood of symptoms, this approach recognizes the patient’s active role in negotiating their experience of reality [76]. To support this, research in schizophrenia should focus more on the experiential dimension of the condition; qualitative methodologies [77] (such as Interpretative Phenomenological Analysis or Consensual Qualitative Research) should be given the importance they deserve to improve current research and better characterize and understand the unique anomalies of schizophrenic consciousness. In light of these distinctions, it becomes imperative to better articulate the epistemological boundaries separating phenomenological inquiry from nosographic classification, as well as therapeutic and translational horizons. The interplay between subjective experience and objective diagnostic criteria underscores the complexity of integrating phenomenological perspectives into clinical practice. This integration demands careful attention to both the limitations and possibilities inherent in translating subjective anomalies into actionable therapeutic strategies and research paradigms.

## Figures and Tables

**Table 1:** From Clinical Symptoms to Images: A Typology of the Clinical Epoché/Reduction. By using this Table, the clinician can move beyond a purely descriptive psychiatry, using descriptions by philosophers to shed light on patients’ experiences and transforming the examination into a diagnostic act of structural “seeing”. By applying these phenomenological analogies, the clinician avoids reducing the mental state examination to a generic diagnostic box. Ultimately, the table empowers the clinician to practice a poetics of recognition, ensuring the patient’s world is met with an ethically grounded and epistemically sophisticated response.

Epoché/Reduction Type	Synthetic Philosophical Definition	Clinical Manifestation	Key Visual/Analogy
**Eidetic**	Suspending common-sense “typifications” to reach the essence of things.	**Delusional Perception:** Objects lose their mundane context and reveal self-referential truths.	**The Torn Veil:** Reality stripped of its usual utility to reveal a “hidden” meaning.
**Transcendental**	Shifting focus from the object “out there” to the internal psychic acts (*noesis*).	**Hyper-reflexivity:** The mind observes its own mechanics; thought becomes an object.	**A Mirror Reflecting a Mirror:** The infinite regress of self-observation.
**Vital Drive**	Switching off the *Lebensdrang* (vital drive) that anchors to reality.	**Adynamic World:** Derealization where the world loses its density and “vital heat.”	**De Chirico’s Square:** Ghostly shadows, statues, and a “halt of the vital rhythm.”
**Readiness-to-hand**	Suspending the pragmatic involvement with things as tools (*pragmata*).	**Loss of Practical Meaning:** Objects appear as purposeless geometric solids.	**A Technical Blueprint:** A room seen as a cold map rather than a lived-in space.

**Table 2:** A Comparative Matrix Bridging Phenomenological Epoché/Reduction and DSM Clinical Symptoms. This Comparative Matrix provides the necessary bridge between abstract phenomenological theory and the concrete requirements of contemporary psychiatric classification. By aligning the various modes of the epoché/reduction with standard DSM-5 symptoms, we can show how a “symptom” is actually the outward manifestation of a profound “reduction” in consciousness.

Phenomenological Mechanism	Clinical Marker (DSM-5/MSE)	Structural Transformation	Clinical Utility: “Why It Matters”
**Bracketing of Familiarity**	**Perplexity/Delusional Mood**	Involuntary “snapping” of the threads that anchor the self to reality; the world becomes enigmatic.	Helps distinguish perplexity from confusion or delirium; identifies the “aura” of early psychosis.
**Eidetic Reduction**	**Delusional Perception (Apophenia)**	Aberrant Salience: Mundane objects lose their common-sense context to reveal self-referential, “hidden” truths.	Validates the patient’s experience as a shift to *hidden meaning* rather than a cognitive impairment improving therapeutic alliance.
**Transcendental Reduction**	**Hyper-reflexivity/Thought Monitoring**	I-splitting: The “invisible” background of thought becomes a strange, “thing-like” object of focal awareness.	Distinguishes obsessive or disorganized thought from a detached monitoring of the “machinery” of the mind.
**Suspension of the Vital Drive**	**Depersonalization/Derealization**	Disembodiment: The world loses its “grip” and density; reality appears as a flat theatrical set.	Shifts the view from “flat affect” to a total loss of “existential grip” or vital anchor in reality result of the world losing its material “reality” and “call to action.”
**Crisis of Intercorporeality**	**Asociality/Withdrawal**	Failure of bodily resonance; social life becomes a “cool game” governed by abstract rules rather than flow.	Helps the clinician understand “social awkwardness” as a structural loss of intuitive self-world interbody attunement, preventing the mislabeling of the patient as “cold.”
**Collapse of the “As-If”**	**Literalism in Delusions**	**Semantic Collapse:** Metaphors lose their symbolic distance and “become flesh” (e.g., a “broken heart” as a physical fracture).	Identifies when a metaphor has become a physical reality, crucial for marking the transition to a literal, fragmented, and ghostly existence.

## Data Availability

Not applicable.
